# Structural Dynamics of Human Argonaute2 and Its Interaction with siRNAs Designed to Target Mutant tdp43

**DOI:** 10.1155/2016/8792814

**Published:** 2016-03-06

**Authors:** Vishwambhar Bhandare, Amutha Ramaswamy

**Affiliations:** Centre for Bioinformatics, Pondicherry University, Pondicherry 605014, India

## Abstract

The human Argonaute2 protein (Ago2) is a key player in RNA interference pathway and small RNA recognition by Ago2 is the crucial step in siRNA mediated gene silencing mechanism. The present study highlights the structural and functional dynamics of human Ago2 and the interaction mechanism of Ago2 with a set of seven siRNAs for the first time. The human Ago2 protein adopts two conformations such as “open” and “close” during the simulation of 25 ns. One of the domains named as PAZ, which is responsible for anchoring the 3′-end of siRNA guide strand, is observed as a highly flexible region. The interaction between Ago2 and siRNA, analyzed using a set of siRNAs (targeting at positions 128, 251, 341, 383, 537, 1113, and 1115 of mRNA) designed to target tdp43 mutants causing Amyotrophic Lateral Sclerosis (ALS) disease, revealed the stable and strong recognition of siRNA by the Ago2 protein during dynamics. Among the studied siRNAs, the siRNA_341_ is identified as a potent siRNA to recognize Ago2 and hence could be used further as a possible siRNA candidate to target the mutant tdp43 protein for the treatment of ALS patients.

## 1. Introduction

The noncoding genes play important roles in regulatory processes and control gene expression via sequence specific interactions [1, 2]. The RNA interference (RNAi) mechanism was first reported in nematode* C. elegans* and later was also reported in various eukaryotes including human [1–4]. In plant, the RNAi phenomenon is referred to as posttranscriptional gene silencing (PTGS), while in fungus it is known as quelling and is studied first in the model organism* Neurospora crassa *[5–7]. Both PTGS and RNAi show similar mechanism in the process of gene silencing like sequence specificity, mRNA degradation, systematic nature, and so forth [8]. The RNAi, PTGS, and quelling phenomena are closely related and hence represent the conserved ancestral process. In plants and invertebrate, the RNAi is very useful in antiviral defense mechanism [9]. In RNAi, either small interfering RNA (siRNA) or microRNA (miRNA) recognizes their target based on sequence complementarity (partial sequence complementary in the case of miRNA, whereas perfect complementarity is adopted by siRNA) with the help of Argonaute (Ago) protein [10, 11].

The siRNA is a duplex RNA (formed by both guide and passenger strands) having 21 to 25 nucleotides in length with two overhangs at both 3′- and 5′-ends and is generated when the double stranded longer RNAs are processed by the enzyme called dicer [10]. Binding of siRNA duplex to RNA Induced Silencing Complex (RISC) promotes duplex unfolding and cleaves the passenger strand by endonuclease. The RNAi mechanism in human was reported first by Elbashir et al. and highlighted the role of siRNA (nucleotides of length 21-22 bp) in effectively degrading the mRNA [12]. The Ago protein assists the guide strand of siRNA to recognize its complementary target strand, which further cleaves the target mRNA by forming the RISC [6]. Various factors such as Guanine and Cytosine (GC) content, three-dimensional conformation of mRNA and siRNA, and stability of siRNA-mRNA complex determine the efficiency of siRNA [13]. Such rationally designed siRNAs have great importance in RNAi or gene therapy due to the dramatic increase in success rate [14]. Grimm and Kay have reported some of the experimentally identified siRNAs that are under clinical trials [15].

The complete mechanism of RNAi is not fully explored [16]. The reported steps involved in RNAi mechanism are as follows: (i) the cleavage of large double stranded RNA into small fragments of length 21–28 bases (siRNA) with the help of RNA nucleases and (ii) the formation of RISC complex (comprising siRNA, mRNA, and Ago), which cleaves the homologous single stranded mRNA. The RNAi/gene therapy is now being used to treat various diseases like cancer, HIV, Hepatitis virus, neurological disorders, and so forth [17–21]. The key player of RNAi machinery is the Ago protein (a major component of RISC), which helps to find the complementary sequence and finally cleaves the target mRNA sequence with the help of siRNA guide strand. Extensive studies on Ago protein from* S. pombe*,* C. elegans*,* D. melanogaster*, and various mammals have been carried out during the past few years [22]. Ago protein is present in various forms such as Ago1, Ago2, Ago3, and Ago4. Both Ago1 and Ago2 in human share about 80% of similarity. Only Ago2 is having slicer activity and Ago1 fails to express this activity due to the lacking of key catalytic residues [23].

The crystal structure of both archaeal and prokaryotic Ago proteins from* Pyrococcus furiosus *and* Aquifex aeolicus*, respectively, has been reported [24, 25]. Recently, the Ago protein characterized from* Thermus thermophilus* and* Rhodobacter spheroids* revealed the gene silencing mechanism in prokaryotes [26, 27]. The structure of archaeal, prokaryotic, and eukaryotic Ago proteins shows almost similar architecture. The bacterial Ago protein has four domains such as N-terminal, PAZ, MID, and PIWI domains. The PAZ domain of bacterial Ago shows similar architecture with that of archaeal one [28]. Recently, a crystal structure of full length human Ago2 protein (PDB ID: 4OLA) has been reported by Schirle and MacRae [29]. Human Ago2 protein shows a bilobed structure with (i) N-terminal (Asp53-Ser139), (ii) PAZ (Pro229-Val347), (iii) MID (Gly445-Pro580), and (iv) catalytic PIWI (Gln581-Ala859) domains. The N-terminal and PAZ domains are connected by linker L1 (Leu140-Gln228), while the PAZ and MID domains are connected by linker L2 (Ala348-Thr444). The PAZ domain, which is similar in the architecture of Ago proteins from various species, plays an important role in holding the 3′-end of siRNA. The key residues that bind with the 3′-end of siRNA are Lys191 and Tyr259. Residues such as Lys252 and Gln276 are also reported to interact with siRNA [24]. The MID domain binds to the 5′-end of siRNA. The PIWI domain plays a major role in RNA cleavage as it possesses slicer activity similar to RNase H [30]. The two Mg^2+^ ions, coordinating the catalytic triad formed by DDH motif in PIWI domain, play a catalytic role in the endonucleolytic cleavage of target mRNA. Mutation at the catalytic residues in active site fails to express slicer activity [31]. In Ago, the Arginine residue, which is populated majorly in PAZ, MID, and PIWI domains, significantly interacts with siRNA [32].

To explore the role of Ago2 in line with RNAi mechanism, the incurable neurological disorder named as Amyotrophic Lateral Sclerosis (ALS) has been considered for this study. ALS is a fatal neurological disorder, involving the large motor neurons of brain and spinal cord. ALS is characterized by the progressive paralysis and subsequent death due to respiratory failure [33]. The disease pathogenesis is unknown and hence no effective treatments have been reported yet [34]. Riluzole is the only drug used to treat ALS, which could only delay the disease progression and death [35]. In ALS, several point mutations have been reported in various proteins like SOD1, FUS, TDP43, Profilin 1, Ubiquilin 2, and Vasolin containing proteins [36–40]. The TDP43 is a RNA binding protein having the molecular weight of 43 kDa and is reported as a majorly identified disease protein in both sporadic and familial ALS [41]. The TDP43, aggregated in neural cells, are linked to several neurological disorders [37]. Various point mutations in TDP43 are proven to cause various neurological disorders like FTLD, Alzheimer, and ALS [39, 42, 43] and are summarized in PROMINE database [44]. The C-terminal glycine rich domain has most of the point mutations causing ALS disease.

Knowledge on the interaction of siRNAs with Ago2 protein gains more importance as they play a vital role in RNAi therapy. Very recently, a systematic analysis has been performed by our group to design and screen possible siRNAs to effectively target the C-terminal region of TDP43. Among the studied siRNAs, few of the identified potential lead candidates (named according to their start position in mRNA and listed in Table 1) have been used in this present analysis to understand both the binding nature and efficiency of siRNAs with Ago2 protein [45]. The selected siRNAs (having high GC content and thermal susceptibility about ~40.14% and ~60.24, resp.) expressed significant binding free energy (−33.41 kcal/mol) in agreement with the experimentally reported siRNAs designed for gastric carcinoma and diseases [46].

Overall, knowledge on Ago2 protein, which potentially guides the siRNA for endonuclease activity, would provide a baseline understanding at the atomic level in regard to the RNAi mechanism for treating ALS disorder. Hence, in the present study, structural dynamics of both free and siRNA bound human Ago2 protein are analyzed using molecular dynamics simulations and the observations are explained in the view of RNAi mechanism. The identified interactions of siRNA, targeting the C-terminal domain of TDP43, with human Ago2 protein would help to explore the mechanism of guide strand recognition by Ago2 in human RNAi.

## 2. Methodology

### 2.1. Modeling the Structure of Human Ago2

The crystal structure of full length human Ago2 protein with a resolution of 2.3 Å was retrieved from Protein Data Bank (PDB ID: 4OLA). The missing loop regions were modeled using Sybyl biopolymer module. The divalent Mg^2+^ metal ions required for the catalytic cleavage of the target mRNA were fixed similar to the reported coordination in* T. thermophilus* Ago protein (PDB: 3HVR) [30] using Discovery Studio 3.1. The modeled Ago2 protein was relaxed for a period of 6 ns using all atom molecular dynamic simulations with restraint only on Mg^2+^ to maintain the metal coordination. The relaxed structure of Ago2 was subjected to (i) qualitative analysis using SAVES server and (ii) secondary structure analysis using Ramachandran plot.

### 2.2. Modeling the Structure of Human Ago2-siRNA Complexes

The complex formation of any ligand with a biomolecule is effectively modeled using docking procedure. In a standard docking protocol, the ligand would identify a comfortable interaction site at the periphery of the biomolecule based on the interaction energy [47, 48].

Modeling of the complex structures of Ago2-siRNA was carefully performed as the single stranded RNA is embedded in between the globular domain formed by the four domains such as NTD, PAZ, MID, and PIWI of Ago2 protein and particularly held by both PAZ and MID domains [31]. As reported in the crystal structure, the standard docking procedure would not allow the siRNA to penetrate inside the globular domain of Ago2 but could identify the interaction sites only at the periphery of Ago2, which is not the case here. Hence, the standard docking procedure was not adopted in modeling Ago2-siRNA complexes.

A crystal structure of Ago2 with guide RNA molecules (PDB ID: 4OLA superseded with 4EI1), reported by Schrile and MacRae [29], was used as the template structure for modeling Ago2-siRNA complexes. In the selected template structure (PDB ID: 4OLA superseded with 4EI1), only the starting eight bases from the 5′-end and last one base from the 3′-end were reported. The structure as well as orientation of complete RNA backbone inside the globular domain of Ago2 was modeled according to several other Ago2 proteins complexed with RNA from various species (PDB IDs: 3DLB, 3HVR, and 1YTU). After modeling the backbone of guide strand RNA inside the Ago2 protein, the siRNA structure has been modeled based upon the required nucleotide sequence by changing only the bases using Accelrys Discovery studio 3.1.

For the present analysis, a set of seven siRNAs (having target positions at 128, 251, 341, 383, 537, 1113, and 1115 in tdp43 gene) designed to target tdp43 mutants causing ALS disease [45] have been designed by modeling the required bases while maintaining the conformation of siRNA backbone as in the Ago2-siRNA template structure. Finally, the required Ago2-siRNA complexes were modeled by first overlapping both structures of Ago2 (the new modeled Ago2 and the Ago2-siRNA template with the newly modeled siRNA) and then the template Ago2 structure was eliminated. The resulting newly modeled Ago2-siRNA complex structures were subjected for energy minimization. At first, only the newly added bases were relaxed with constraints on both RNA backbone and Ago2 to void off the steric interactions arising from modeling, followed by the relaxation of the entire molecular system (Ago2-siRNA) without any constraints. The optimized conformations of Ago2-siRNA complexes were used to explore guide strand recognition by Ago2 using molecular dynamics study.

### 2.3. Molecular Dynamics Simulation

All atom molecular dynamic simulations were carried out for the modeled human Ago2 and Ago2-siRNA complexes using Gromacs 4.5.5 package [49] with AMBER99-ILDN force field [50]. In order to understand the dynamic behavior of various domain motions in human Ago2, molecular dynamics simulation was performed for a period of 25 ns. The Ago2 protein was placed in the center of a cubic box of dimensions extending about 10 Å from the extents of the protein. A water model of TIP3P was used and the complete system was neutralized using Cl^−^ counter ions. All the systems were simulated with periodic boundary conditions and were minimized for 50,000 cycles using steepest descent followed by conjugate gradient algorithms. The system was equilibrated for a period of 100 ps using NVT and NPT ensembles, respectively. During simulation, the temperature was maintained at 300 K using a coupling method called Berendsen thermostat to regulate the temperature [51], and the pressure of the system was maintained at 1 bar using Parrinello-Rahman barostat [52]. The long range electrostatic interactions and covalent bonds involving H-atoms were treated with Particle Mesh Ewald (PME) method and LINCS [53] algorithm, respectively. For the short range interactions, cut-off values for van der Waals and coulombs were adopted as 10 Å, respectively. The production MD was recorded at every 2 ps over the simulation period of 25 ns.

The Ago2-siRNA complexes were also subjected to similar protocol of simulation and were analyzed for a period of 15 ns. The parameters explaining the conformational stability such as RMSD, RMSF, and *R*_*g*_ have been measured and were analyzed using the tool Grace 6.0. The three-dimensional structures were visualized by UCSF Chimera 1.9 [54] and the domain motions in Ago2 were explained using DynDom analysis [55]. The intra- as well as interdomain motions/correlations were analyzed using the Amber tool 14.0 [56].

### 2.4. Cross Correlation Analysis

The concerted motions exerted by Ago2 protein during molecular dynamics simulation have been analyzed using the cross correlations expressed by the time dependent fluctuations of C_*α*_-atoms. The cross correlation is given by

(1) $$\begin{array}{c} Cij=\frac{c\left(ij\right)}{{\left[c\left(i,i\right)c\left(j,j\right)\right]}^{1/2}}=\frac{\langle {\Delta r}_{i}\cdot {\Delta r}_{j}\rangle }{{\langle \Delta r_{i}^{2}\rangle }^{1/2}{\langle \Delta r_{j}^{2}\rangle }^{1/2}}, \end{array}$$

where Δ*r*_*i*_ and Δ*r*_*j*_ are the displacements from the mean position of *i*th and *j*th of C_*α*_-atoms averaged over the trajectory [57, 58]. The deviation between −1 and 1 indicates the amplitude of correlation, where *C*(*i*, *j*) = 1 represents the complete correlated motion (i.e., motion in the same direction) and *C*(*i*, *j*) = −1 represents the completely anticorrelated motions (i.e., motion in the opposite direction).

### 2.5. Binding Free Energy Calculation

The calculation of binding free energy (Δ*G*_bind_) of complex between human Ago2 and selected siRNAs was performed as follows:

(2) $$\begin{array}{c} \Delta G_{bind}=G^{Ago2\text{-}siRNA}-G^{Ago2}-G^{siRNA}, \end{array}$$

where *G* ^Ago2-siRNA^, *G* ^Ago2^, and *G* ^siRNA^ are free energies of Ago2-siRNA complex, Ago2, and siRNA, respectively. The individual components mentioned above are calculated as given below:

(3) G=EMM+GPB+GSA−TS,EMM=Evdw+Eelec+Einit.

*E*_MM_ is molecular mechanical energy and *G*_PB_ and *G*_SA_ are polar and nonpolar terms from implicit calculation. The recently published tool “g_mmpbsa” was used for these calculations [59]. During the stable dynamics after 3 ns, frames at 1 ns of interval were taken from the trajectory. For the implicit solvation analysis, dielectric constants of 2 and 80 are used for solute and solvent, respectively. The polar solvation energy was calculated using Poisson-Boltzmann (PB) equation as below:

(4) ∇εr∇·ϕr−εrkr2sin⁡hϕr+4πρfrkT=0.

In PB equation, *ϕ*(*r*) is electrostatic potential, *ε*(*r*) is dielectric constant, *ρ* ^*f*^(*r*) is the fixed charge density, and *k* ^2^ is the reciprocal of Debye length independent of ionic strength of solution.

The most widely used solvent accessible surface area, SASA model, was used for nonpolar energy calculation as given below:

(5) $$\begin{array}{c} G_{SA}=\gamma A+b, \end{array}$$

where “*γ*” is the surface tension coefficient of the solvent and “*b*” is the fitting parameter. The radius for surface tension value and solvent accessible surface area were set to 0.0226 and 1.4 Å, respectively.

## 3. Results and Discussion

Several crystal structures of Ago proteins with guide strand from various species including* T. thermophilus* (3DLH/3F73) and* A. aeolicus* (1YVU) are reported [21, 25, 30]. Interaction of siRNA with human Ago2 gains importance due to the increased success rate of siRNA assisted gene silencing therapy. The dynamic behavior of human Ago2 in presence of siRNA is not yet explored due to the lack of human Ago2 protein structure. Recently, the crystal structure of human Ago2 has been reported by Schrile and MacRae [29]. In the present study, the interaction of human Ago2 with siRNA is studied for the first time using seven siRNAs, which are designed to target the tdp43 mutants causing ALS disease [45]. The structure of modeled Ago2 protein was relaxed for a period of 6 ns to void off steric clashes and was further simulated for a period of 25 ns to understand its time dependent structural dynamics. A systematic analysis on the interaction of Ago2 protein with the selected siRNAs was performed by simulating the Ago2-siRNA complexes for a period of 15 ns. The specificity of all siRNAs towards the Ago2 protein was estimated by calculating the binding free energy. The siRNA showing strong binding affinity (siRNA_341_) with Ago2 was further simulated for a period of 25 ns to explore the binding mechanism of siRNA guide strand with Ago2 protein.

### 3.1. Dynamics of Free Ago2

**Figure 1:**
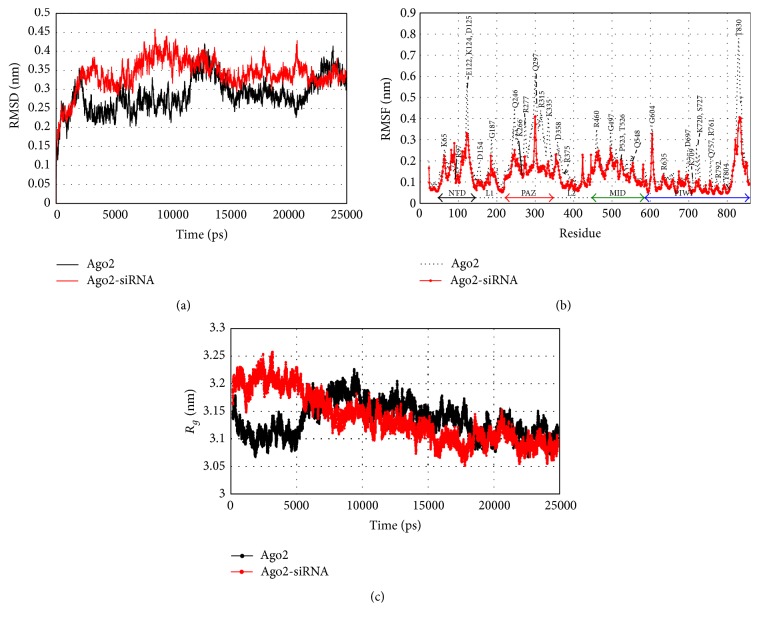
Variation in RMSD (a), RMSF (b), and *R*_*g*_ (c) values for both free (black lines) and siRNA_341_ bound (red lines) Ago2 protein calculated during dynamics.

As the first phase of study, the dynamics of Ago2 protein was observed for a period of 25 ns to understand its structural dynamics. The variation in RMSD observed during simulation is shown in Figure 1(a). After a simulation of 2.5 ns, the Ago2 protein expressed stable dynamics with RMSD value less than 3 Å (black line in Figure 1(a)). These stable dynamics also revealed another short lived conformational state with higher RMSD value (about 3.75 Å) during 12.5–14.5 and 22.5–24.5 ns of simulation period. Representative conformations at these RMSD values were extracted to get insight into the structural states of Ago2 protein during dynamics. Superimposition of these two structures revealed significant changes in the orientation of PAZ domain and disclosed the existence of Ago2 protein in both “open” and “close” conformations during dynamics. In the “open” conformation, both PAZ and NTD domains come closer, while the MID and PAZ domains move away from each other, whereas in the “close” conformation, the flexible PAZ domain moves closer to the MID domain in order to facilitate the binding of guide strand of siRNA. As a consequence, both MID and PAZ domains of Ago2 efficiently anchor the 5′- and 3′-ends of siRNA, respectively. Hence, the observed “open” and “close” conformations of Ago2 mediated by the movement of PAZ domain reveal its dynamic role in anchoring the single stranded guide siRNA (of length 21–28) between the PAZ and MID domains of Ago2 protein.

### 3.2. Dynamics of Ago2-siRNA Complexes

All atom simulations have also been performed on siRNA bound Ago2 complexes (using the siRNAs listed in Table 1) to understand both the nature of siRNA binding with Ago2 protein and the efficiency of siRNAs designed for ALS disease. The binding of siRNA with Ago2 is analyzed by monitoring the conformational stability of Ago2-siRNA complexes for a period of 15 ns. All the Ago2-siRNA complexes are well stabilized within the simulation period of 5 ns with RMSD values ranging between 1.8 and 4 Å (Supplementary Figure 1A in Supplementary Material available online at http://dx.doi.org/10.1155/2016/8792814). Among the studied complexes, the Ago2-siRNA_251_ and Ago-siRNA_341_ express the least and highest RMSD (1.8 Å and 3.7 Å, resp.), while the rest of the complexes fluctuate between 2.5 and 3.5 Å. The root mean square fluctuations of the C_*α*_-atoms in all Ago2-siRNA complexes have been analyzed. All these complexes expressed similar pattern of fluctuation (Supplementary Figure 1B). The residues of Ago2-siRNA_251,383,537,1113,1115_ complexes show higher fluctuations than other studied complexes. It is also seen that the flexible dynamics of Ago2 are highly driven by the fluctuation of the PAZ domain of Ago2.

The structural compactness calculated for all Ago2-siRNA complexes converges between 3.08 and 3.2 nm^2^ and indicates the stable fluctuation expressed by the domains of Ago2 during dynamics (Supplementary Figure 1C). Among the studied complexes, one of the complexes, formed between siRNA_341_ and Ago2 protein, revealed a highly stable dynamics over other complex systems and hence the conformational evolution of this Ago2-siRNA_341_ complex was compared with the dynamics of free Ago2 protein (red line in Figure 1(a)). Analysis also reveals that initially the Ago2 exists in an open conformation (with the RMSD value of 2.5 Å) and after 3 ns, Ago2 adopts the close conformation (with the RMSD value of 4 Å) to facilitate binding with siRNA. Both of these open and close conformations are already emphasized by the free Ago2 protein during simulation. Thus, the RMSD analysis of free and siRNA bound Ago2 proteins highlights the existence of open and close conformations of Ago2 protein as well as the effective siRNA binding by the close conformation of Ago2 protein.

The observed fluctuation of C_*α*_-atoms in Ago2 during dynamics has been depicted in Figure 1(b). The free Ago2 protein (black dotted line) exists in a highly flexible state, when compared to the siRNA bound Ago2 (red continuous line). During simulation, the negatively charged (Asp, Glu), polar (Gln, Ser,), and charged (Arg, Lys) residues reveal dynamic nature, whereas the hydrophobic residues do not participate in high amplitude fluctuation due to their buried nature of side chains towards the interior of the Ago2 protein. The regions expressing higher atomic fluctuations are contributing significantly to the dynamics of Ago2 and are (i) PAZ domain, (ii) Glu122, Lys124, and Asp125 residues of NTD, and (iii) Thr830 from the C-terminal PIWI domain. When siRNA binds to Ago2, both of its 3′- and 5′-ends are anchored by the PAZ and MID domains, respectively, via H-bonding interactions and the regions of siRNA between these ends are held by both NTD and PIWI domains. These interactions are modulated by the close conformation of Ago2, which is mainly defined by the reorientation of PAZ domain towards the MID domain. It is observed that the dynamics of the residues of Ago2 interacting with siRNA are conserved in all the studied systems, and hence similar pattern of RMSF fluctuation has been observed (Supplementary Figure 1B). From RMSF, it is clear that both N-terminal and PAZ domains express a maximum fluctuation of 5 Å, whereas both MID and PIWI domains show very less fluctuations except few residues at the terminal loop region. The free dynamics/fluctuation of Ago2 protein is significantly confined by the inter-H-bonding interactions existing between Ago2 and siRNA. Explicitly, the close conformation of Ago2 in the complex form is highly stabilized by several H-bonds mediated by the residues of MID domain such as Pro523, Thr526, Gln548, and Lys355 with siRNA. In this bound form, the catalytic residues DDH (597, 669, and 807, resp.), which cleave the target guide strand of mRNA, are significantly stable. Overall, it is observed that Ago2 adopts a close conformation to anchor the siRNA at the binding cleft.

**Figure 2:**
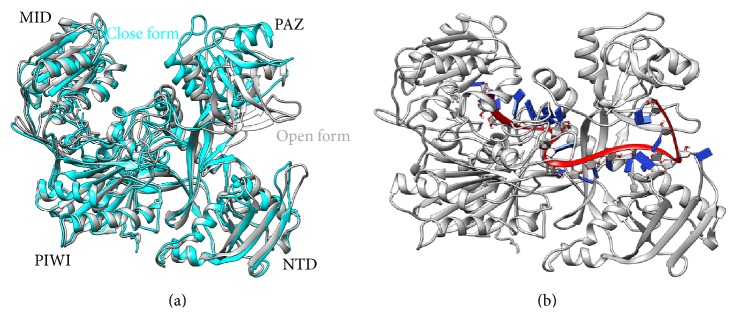
Three-dimensional structure of “open” and “close” conformations observed during the dynamics of free Ago2 (a) and the siRNA_341_ guide strand recognition by Ago2 (b).

The structural compactness of Ago2 protein in both free and complexed form was monitored during simulation by calculating the “*R*_*g*_” value.  Figure 1(c) depicts the variation in the *R*_*g*_ value of Ago2 protein in the absence and presence of siRNA (i.e., free Ago2 in black lines and siRNA_341_ bound Ago2 in red lines) and reveals stable dynamics after 15 ns. The stable dynamics of all Ago2-siRNA complexes are reinforced by the stable variation in the *R*_*g*_ value (3.11 ± 0.4 nm). Among the selected siRNAs, the siRNA_341_ is identified as the strongly bound substrate with Ago2 (converging at 3.02 nm of *R*_*g*_). For a comparison analysis, the *R*_*g*_ value of Ago2, in the presence and absence of siRNA_341_, is plotted in Figure 1(c) (red and black lines, resp.). Ago2 in both free and siRNA_341_ bound forms shows stable dynamics after 17 ns with *R*_*g*_ less than 3.1 nm (Supplementary Figure 1C). The open and close conformations of Ago2 are depicted in Figure 2(a) and the effective binding of siRNA_341_ by the close conformation of Ago2 protein is shown in Figure 2(b).

The domains such as N-terminal, PAZ, MID, and PIWI also reveal stable dynamics during simulation and are shown in Supplementary Figure 2. The spatial arrangement of these stable domains in Ago2 protein was analyzed by calculating the interdomain distance using a representative atom from the respective stable domains (S131 and V165 from NTD, L328 from PAZ, A452 from MID, and A613 and A596 from PIWI) and is shown in Supplementary Figure 3. It is observed that the MID domain maintains the interdomain distance with both PIWI and NTD domains, whereas the instantaneous positional change in PAZ domain results in a fluctuation as well as decrease in *R*_*g*_ values.

Among the studied complexes, the complex Ago2-siRNA_341_ is identified as the most stable complex as evidenced by RMSD, RMSF, and *R*_*g*_ analyses (Figures 1 and S1). Most of the complexes are held stable by several strong H-bonding interactions (about 25 to 40) of distance less than 3 Å. A 2D representation of Ago2-siRNA interactions is analyzed using NUCPLOT software [60] and the observed interactions are depicted in Supplementary Figure 4.  Table 2 lists the existing strong interactions between human Ago2 and siRNAs, which are mandatory for efficient RNAi/gene silencing.

### 3.3. Conformational Changes in the Secondary Structure of Ago2 in Free and Bound Form

The changes observed in the secondary structure of Ago2 in free and siRNA bound forms during simulation have been analyzed using DSSP program [61]. Supplementary Figure 5 depicts the two-dimensional representation of the changes observed in the secondary structure of free Ago2 and Ago2-siRNA_341_ complex during simulation. In this 2D representation, the red, blue, purple, grey, green, and yellow colors represent the *β*-sheet, *α-*helix, *π*-helix, “3_10_” helix, bend, and turn, respectively.

The structure of free Ago2 undergoes a relaxation mechanism in which its secondary structure settles down in a tender fashion. Some of the observed structural relaxations are discussed in this paragraph. The helical nature of “*α*” helices at N-terminal is slightly distorted and adopts the turn conformation, whereas the antiparallel “*β*”-sheets remain unchanged. The N-terminal helix formed by residues between Arg68 and His81 in free Ago2 protein loses its helicity in the close conformation of Ago2 while retaining its helicity intact in open conformation. The PAZ domain plays a key role in anchoring the 3′-end of guide strand, and the “*β*”-sheets in this flexible domain expressed a moderate structural disturbance. The 3_10_ helix formed by Thr307-Asp314 residues showed minor changes without altering its helicity during simulation. In the open conformation of Ago2, two short lived 3_10_-helices are formed by the residues (i) Pro229-Val237 and (ii) Thr251-Ile262 of the MID domain that holds the 5′-end of the guide strand. The MID domain also expresses the formation of a “*π*” helix by the residues Pro527-Thr538, which vanishes after 17 ns. The C-terminal PIWI domain of Ago2 undergoes a major conformational change during simulation.

The helicity of two short C-terminal helices such as His839-Gln847 and Gln850-Arg854 in the PIWI domain is altered during the dynamics of open conformation of Ago2. One of these helices, Gln850-Arg854, regains its helical conformation from 17 ns onwards. The bend located at the terminal region of PIWI domain (observed from DSSP analysis) disappears after 10 ns of simulation. The 3_10_-helix present at the terminal PIWI domain undergoes fluctuation and changes its conformation into *α*-helix at the end of simulation. The movement of PAZ domain towards mid domain is responsible for this conformational change. PIWI domain has a catalytic triad (DDH) and these conformational changes are necessary to promote the orientation of the target mRNA with which the complementary siRNA would pair and facilitate the catalytic cleavage by nucleophilic attack.

Similarly, the secondary structure analysis has also been performed for the Ago2 protein in Ago2-siRNA_341_ complex. The residues of Ago2 involved in interactions with siRNA expressed stable dynamics of the secondary structure compared to those of free Ago2. The N-terminal helix formed by the residues Arg68-His81 is reduced for a short period of time (within 5-6 ns) to Val70-His81 and thereafter maintains its helicity throughout the simulation. In the bound form of Ago2 (i.e., Ago2-siRNA_341_) the helix formed between the residues Arg-68-His81 is changed to Arg69-Gln80. The Arg68 residue is actively engaged in the electrostatic interaction with siRNA. Neighboring residues like Glu64, Lys65, and Pro67 are also participating in the nonbonded interactions (i.e., Vdw and electrostatic interactions) with siRNA. The interactions of these residues with siRNAs disturb the nature of this helix. A single short lived 3_10_ helix is formed by the residues Pro585-Gln588 in the catalytic PIWI domain. The helix formed by the residues Pro373-Arg384 from linker L2 is unfolded during the opening and closing motions of both PAZ and MID domains in bound Ago2, while this helix retain its helicity during simulation in free Ago2.

In summary, secondary structure of Ago2 in both open and close conformations retains its structural stability during the simulation, though there are minor conformational changes. The observed conformational changes in the secondary structures of MID and PAZ domains of Ago2 are attributed to the flexible nature of the conformation of Ago2 protein to sense the guide strand of siRNA for the stable binding with target mRNA.

### 3.4. The Interdomain Interactions Stabilized by H-Bonds

The analysis of interdomain H-bonds revealed strong H-bonding interactions between the domains of Ago2. Ago2 expresses different pattern of interdomain H-bonds in both “open” and “close” conformations. Throughout the simulation, the MID and PIWI domains of free Ago2 are stabilized by about 28 interdomain H-bonding interactions. The N-terminal and PAZ domains show only 8 interdomain H-bonds. The interaction between N-terminal and PAZ domains is further decreased, when the PAZ domain moves towards MID domain (i.e., away from N-terminal domain) to attain the close conformation of Ago2. Hence, the short lived close conformation of Ago2 is governed by the movement of flexible PAZ domain. The interdomain distance calculated using the representative residue from each domain gives insight into the spatial distribution of all the domains of Ago2 during simulation and is shown in Supplementary Figure 3. The distance between (i) MID and PIWI domains (shown in black line) and (ii) MID and N-terminal domains (shown in red line) remains stable throughout the simulation (Supplementary Figure 3). As the flexible PAZ domain mediates both the open and close conformations of Ago2 protein (which facilitates further anchoring of guide siRNA between PAZ and MID domains), the distance between PAZ and NTD fluctuates during simulation. The observed flexible nature of PAZ domain has also been reported by various experimental and theoretical studies [21, 28, 62–65].

### 3.5. Cross Correlation Map

**Figure 3:**
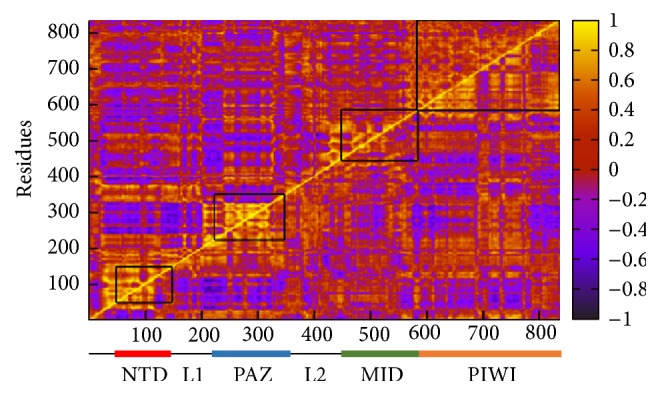
The cross correlation between the residues of Ago2 protein in both free (upper diagonal) and siRNA_341_ bound (lower diagonal) states. The cooperative and anticooperative motions range from −1 to 1 (dark blue to amber color).

The correlation map representing the intra- as well as interdomain motions has been generated from the stable trajectory observed at the last 5 ns of simulation using Amber tool 14.0 [66, 67]. A comparative analysis on the domain motions of free as well as siRNA_341_ bound Ago2 protein has been performed to understand the binding mechanism of siRNA with Ago2.  Figure 3 depicts the cross correlation motion observed with free (upper diagonal) and siRNA_341_ bound Ago2 protein (lower diagonal). The diagonal line (amber color) expresses the strong autocorrelation between Ago2 residues. The spectrum of correlated to anticorrelated motions is scaled from amber to blue, respectively.

In free Ago2, the domains including NTD, PAZ, and MID express strong autocorrelation, whereas the C-terminal PIWI domain expresses a comparatively lesser autocorrelation. The regions NTD and linker L1 (Ser139-Pro229) and few residues from L2 (i.e., Val347-Ser380) are involved in a concerted motion between themselves. Both MID and PIWI domains are involved in a less amplitude cooperative motion. The catalytic residues Asp597, Asp669, and His807 (DDH motif) ensure a conserved evolution during simulation. At the same time, a part of PAZ domain (formed by residues Val230–Val330) is not interactive with NTD. Overall, the free Ago2 expresses significant cooperative as well as anticooperative domain motions during dynamics and the anticooperative motion observed between PAZ and NTD domains delocalizes the PAZ domain closer to the MID domain and facilitates the transition to “close” state of Ago2.

When siRNA binds to Ago2, (i) the degree of autocorrelation in NTD is decreased, and the linker L1 moves along with PIWI to bring the seed region closer to catalytic triad, (ii) a high amplitude autocorrelation is observed with PAZ domain, when compared to both MID and PIWI, and (iii) the PAZ and NTD move opposite to each other; that is, the flexible PAZ domain moves towards MID domain to accommodate the longer guide strand of siRNA and such closure movement of PAZ domain towards MID domain is responsible for the anticooperative motion of PAZ domain with NTD. The helix (formed by Pro527-Asp537) of MID domain (where 5′-end of siRNA is anchored) shows strong correlation with PAZ domain that holds the 3′-end of siRNA. Overall, both MID and PAZ domains are involved in anticooperative/scissoring motion with which the Ago2 adopts either open or close conformations. Binding of siRNA inhibits this anticooperative/scissoring motion of these domains and establishes a cooperative motion between them in order to facilitate its binding to Ago2 and hence stabilizes the complex at close conformation (Supplementary Figure 6).

### 3.6. Directions of Domain Motions

**Figure 4:**
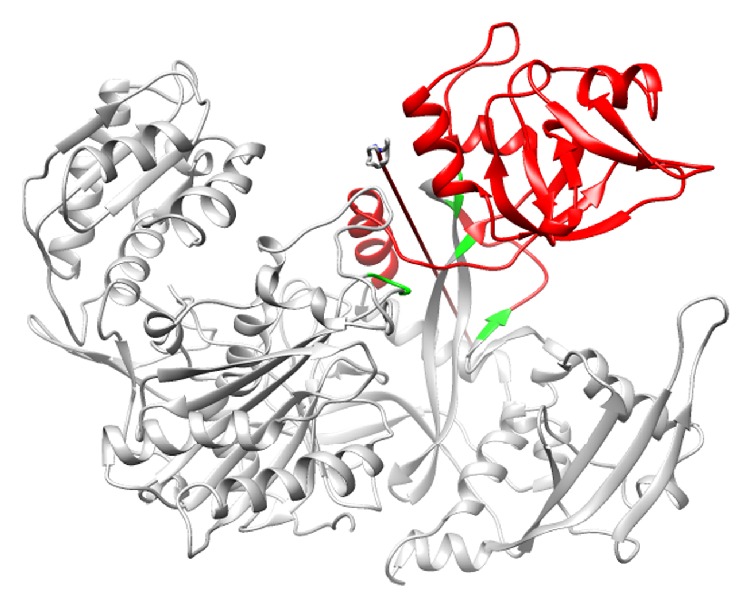
The functional dynamics of free Ago2 protein analyzed using DynDom. The dynamic PAZ domain is colored in red, for which the residues colored in green act as hinge domain. The arrow indicates the hinge axis for the rotation of PAZ domain.

Protein domain motion analysis was performed for the representative average frames extracted from both open (within 12-13 ns) and close (within 18-19 ns) conformations of Ago2 protein using DynDom server. The free Ago2 expresses two domains such as (i) the moving PAZ domain (from Tyr225 to Arg351) for which the residues Phe224-Tyr225 and Arg351-Cys352 act as hinge and (ii) the fixed domain formed by N-terminal, MID, and PIWI domains. In Figure 4, the moving domain, fixed domain, and hinges are shown in red, grey, and green color, respectively. Only the PAZ domain expresses a very high closure property (99.2%) mediated by both translational (−0.2 Å) and rotational motions (13.9°) towards the MID domain with respect to the hinge residues (Figure 4). When siRNA binds to Ago2, the observed domain motions are arrested as both PAZ and MID domains anchor both 3′- and 5′-ends of siRNA, respectively.

### 3.7. Conformation of siRNA

The conformation of single stranded siRNA in the presence of Ago2 has great significance in RNAi therapy and was analyzed for all Ago2-siRNA complexes using DSSR software [68, 69]. Analysis on backbone angles (such as alpha (*α*), beta (*β*), gamma (*γ*), delta (*δ*), epsilon (*ε*), and zeta (*ξ*)) of the single stranded siRNA reveals that, after the complexation with Ago2, the guide strand siRNA is stabilized in A-conformation (in agreement with the reported reference values), which is reported as one of the necessary factors for an efficient RNAi/gene silencing [70]. The pseudo-torsion analysis also ensured the existence of siRNA in “A-form.” Such binary Ago2-siRNA complex recognizes and cleaves the target mRNA to block the expression of mutant tdp43 protein.

### 3.8. Site Specific Interactions of siRNA in Human Ago2

It is observed that binding of siRNA with Ago2 is highly mediated by H-bonding interactions. The interaction between siRNA and Ago2 was analyzed by considering the three fragments of RNA such as base, sugar, and phosphate group and the interacting sites are tabulated in Table 2. It is observed that siRNA interacts (via H-bonding) with Ago2 via the backbone phosphates and bases, out of which the backbone phosphate groups are identified as the majorly contributing sites for interaction.

The nonbonded contacts such as Van der Waals and electrostatics interactions majorly contribute to form a stronger interaction between the Ago2 and siRNAs in all studied complexes. In addition, several salt bridges are also observed between the positively charged residues (i.e., Arginine and Lysine) and the negatively charged phosphate group of siRNA (such as Lys533, Lys566, Arg792, Arg710, Arg277, and Lys65) in Ago2-siRNA complexes. All these bonded and nonbonded interactions are shown in Supplementary Figure 4. Mainly, residues such as Lysine, Arginine, Aspartate, and Glutamine present at the PAZ domain form significant H-bonds with the 3′-end of siRNA guide strand. These observed interacting residues are in agreement with the reported siRNA binding residues [24]. The PAZ domain residue, Arg277, is conserved and plays a key role in recognizing the guide strand and also influences the orientation of the phosphodiester backbone of guide strand. Majority of the studied Ago2-siRNA complexes disclose a common interacting pattern with Ago2 and the H-bonding interactions observed in all Ago2-siRNA complexes are listed in Table 2. All siRNAs express the common interacting residues from MID and PAZ domain such as Gln548, Thr526, Lys566, Lys266, and Arg277, respectively.

The 5′- and 3′-terminal bases of siRNA are involved in maximum number of H-bonding interactions as well as other nonbonded interactions (such as Vdw and electrostatics) and allow the rest of bases to interact freely for sensing mRNA in a complementary fashion. The flexibility of PAZ domain is responsible for the release of 3′-end of guide strand. The dynamic PAZ domain also facilitates the access of siRNA bases to the target mRNA. Superimposition of all siRNAs to depict their positioning inside Ago2 protein after complexation is shown in Supplementary Figure 7.

### 3.9. Binding Free Energy

Binding efficiency of the selected siRNAs with Ago2 was analyzed by calculating the relative binding free energy using MMPBSA method [71]. The calculated binding free energy including the energy components such as van der Waals, electrostatic, polar, and nonpolar solvation energies is listed in Table 3. For all complexes, the van der Waals and electrostatic energy components vary as −235.64 ± 20.73 and −5546.97 ± 245.32 kcal/mol, respectively. The solvation energy parameters such as polar and nonpolar (1139.15 ± 144.51 and −28.71 ± 2.06 kcal/mol, resp.) also significantly influence the binding of siRNA. Among the studied siRNAs, the siRNA_341_ shows more affinity towards Ago2 (−4819.66 kcal/mol) whereas the siRNA_1115_ shows less affinity (−4474.58 kcal/mol) and highlights the sequence specific binding affinity of siRNA towards Ago2.

It is interesting to observe a highly favorable (most negative) binding energy for siRNA_341_ despite the observed high RMSD during dynamics. Unlike other siRNAs, the 7–18 nucleosides of siRNA_341_ are positioned closer to the linker region of both N-terminal and PAZ domains and establish stronger interactions with Ago2. Such strong interactions facilitate further strong affinity towards Ago2 and hence promote least binding energy for this complex. From the simulated data, the siRNA_341_ is identified as the efficient siRNA among the studied siRNAs. Such stronger as well as favorable interactions between Ago2 and siRNA are responsible for successful RNA interference.

### 3.10. Validation of These Modeled siRNAs with the Experimentally Reported siRNAs

Two experimentally validated siRNAs, which are evaluated to target the ICAM2 and GRK genes, were used to form complex with Ago2 protein and are compared to the complexes of Ago2 with the rationally designed siRNAs to target the tdp43 mutants. The complexes of these two experimentally proven siRNAs with Ago2 protein expressed stable dynamics similar to those of Ago2 complexes with the siRNAs designed to target the tdp43 mutants. And the interaction patterns expressed by the experimentally proven siRNAs with Ago2 are significantly comparable to that of siRNAs used for the present study. The potential of newly designed siRNAs in sensing the Ago2 protein was analyzed in comparison with these experimentally reported siRNAs, by performing binding free energy analysis. The siRNAs designed to target ICAM2 and GRK genes express the binding free energy values −4846.78 and −4610.42 kcal/mol, respectively, with Ago2 protein, which are in the similar range of siRNAs designed to target mutant tdp43. In the complexes of Ago2 with both siRNAs (designed to target mutant tdp43 and target ICAM2 and GRK genes), the energy components such as van der Waals, electrostatic, and solvation parameters that explicitly define the efficiency of siRNA to interact with Ago2 also vary in agreement with each other. As the binding free energy for the experimentally reported siRNAs and rationally designed siRNAs falls in the same range, we could infer that the rationally identified siRNAs designed to target the mutant tdp43 are also expected to perform equally well in gene silencing phenomenon as that of experimentally reported siRNAs.

## 4. Conclusion

The human Ago2 protein plays a key role in the sequence specific cleavage of target mRNA in RNAi pathway. The dynamics of complete human Ago2 protein have been studied for the first time, to understand (i) the global dynamics of Ago2 and the role of individual domains for its functional dynamics and (ii) the interactions of Ago2 with siRNAs (which are designed to target tdp43 mutants causing ALS disease) with a view to highlight mRNA recognition by siRNA. The molecular dynamics study performed for a period of 25 ns revealed the conformational transition of Ago2 between open and close conformations promoted by the flexible PAZ domain to accommodate the siRNAs (which range in length within 20–28 base pairs) between the MID and PAZ domains. It was also ensured by the molecular dynamics of complex Ago2-siRNA that the close conformation promotes stable siRNA binding. It is also observed that the catalytic DDH triad from the PIWI domain moves towards the seed region of the target to facilitate further cleavage of target mRNA at the seed region (10-11 bases). Dynamics analysis on the interaction of Ago2 with these selected 7 siRNAs (targeting at positions 128, 251, 341, 383, 537, 1113, and 1115 of mRNA) reveals that the studied siRNAs have significant specificity towards Ago2 and form stable complex.

In the complex, the 3′-end of siRNA is held by the PAZ domain by forming H-bonds with Gln297, Arg315, Arg277, and Asp125 residues of Ago2, whereas the 5′-end interacts with Gln548, Pro523, Thr526, Asn551, Lys566, Asn562, Gln545, Tyr529, Tye790, and Arg792 residues of MID domain via stable H-bonds. The rest of siRNA nucleotides positioned on the inner cleft (formed by all N-terminal, PAZ, PIWI, and MID domains) are freely available to sense mRNA during RNAi. The binding free energy analysis reveals siRNA_341_ as the most compatible siRNA among the studied ones.

In summary, knowledge on the structure and dynamics of human Ago2 protein and its interactions with siRNA would promote further understanding of RNAi mechanism in human, which is not yet explored significantly. The lack of efficient treatment for the neurological ALS disorder further necessitates the identification of alternatives to the existing treatment. Success rate of gene therapy, especially using rational design of siRNA, has increased drastically in the past few years and has no/fewer side effects. Hence, the present observations highlight the possible potent/more efficient siRNAs against tdp43 mutants in RNAi mechanism.

## Supplementary Material

Supplementary material contains total 7 figures which are highlighted in the manuscript. Supplementary Figures 1 and 2 represent the parameters explaining the structural stability observed during simulations. Supplementary Figures 3 and 4 show the inter domain distance in free Ago2 and site specific interactions between Ago2 and siRNA, respectively. Changes in the secondary structures are given in the supplementary Figure 5. Supplementary Figures 6 and 7 represent the three dimensional conformations.

## Figures and Tables

**Table 1:** List of rationally designed and experimentally proven (superscripted) siRNAs (having 21 nucleotides) used in this study.

S. number	siRNA	Sequences	GC content in %
1	128	UGAGACACUGGAUUCCUGUUU	43
2	251	UGGAUGAGACAGAUGCUUCUU	43
3	341	CAACCGAACAGGACCUGAAUU	48
4	383	ACCAUAAGAACUUCUCCAAUU	33
5	537	UCAUCUUGGCUUUGCUUAGAA	38
6	1113	CUCUUAUAGUGGCUCUGACUU	43
7	1115	UUAGAGCCACUAUAAGAGUUU	33
8	ICAM2^exp⁡^	CUCUAUAACCGCCAGCGGAUU	52
9	GRK^exp⁡^	AACUGCCUGAAGAGACGUCUU	48

**Table 2:** List of H-bonding interactions observed in Ago2-siRNA complexes. The superscripts “B” and “P” represent the interaction of Ago2 with base and phosphate groups of siRNA, respectively.

128	R	251	R	341	R	383	R	537	R	1113	R	1115	R
U	P523^B^, T526^B^, Q548^B^, N551^P^	U	T526^B^	C	Q548^P^	A		U	K355^B^, Q548^P^	C	Q548^P^	U	
G		G		A	R792^P^	C	K550^B^ K566^P^, R792^P^	C	T559^B^, K566^B^ Y529^P^	U	R792^P^	U	N551^B^, Y529^P^, Q545^P^
A	Y790^P^, R792^P^	G	Y790^P^, R792^P^	A	R792^B^	C	Y790^P^	A	Y790^P^, R792^P^	C	Y790^P^, R792^P^	A	Y790^P^, R792^P^
G	S798^P^	A	S798^P^, Y804^P^	C		A	S798^P^	U	S798^P^, Y804^P^	U	R795^B^ V797^P^, S798^P^, Y804^P^	G	S798^P^, Y804^P^
A	K709^P^, R761^P^	U	K709^P^, R761^P^	C		U	Q757^B^ K709^P^	C	K709^P^	U		A	K709^P^
C	R761^P^	G	R375^P^, R761^P^	G	R761^P^	A	R761^P^	U	R761^P^	A	R761^P^	G	R761^P^
A	R761^P^	A	R761^P^	A	R761^P^	A		U		U	R761^P^	C	R351^P^
C		G	R710^P^	A	R710^P^	G		G	R710^P^	A	R710^P^	C	
U	R635^P^, R710^P^	A	R635^P^	C		A	R710^P^	G	C352^P^ R635^P^	G		A	
G		C	R351^B^	A	H634^B^ R635^P^	A	R710^P^	C	R710^P^	U	H634^B^ R635^P^	C	
G	R179^P^	A		G	R351^P^	C	R635^P^, S672^P^	U	R179^P^	G	R710^P^ S672^P^	U	
A		G	R97^P^	G	R97^P^	U		U	R351^P^	G	R97^P^	A	
U		A	R351^P^	A	K65^B^	U	Q675^B^	U		C		U	
U		U	K266	C		C		G	K266^P^	U	K266	A	
C	K65^B^	G	K266, K278	C		U		C	K65^B^ K278^P^	C		A	
C	K266^P^, R280^P^	C	D64^B^, K65^B^	U	D125^B^	C		U	K124^B^	U	D125^B^	G	
U		U	R280^B^	G	D122^B^ R277^P^	C	R277^P^	U		G		A	
G	D125^B^	U		A		A		A		A		G	
U	R277^P^	C		A	R315^P^	A		G		C		U	R277^B^
U	Q297^B^	U	K335^B^	U		U		A		U		U	

**Table 3:** Binding free energy of the rationally designed and experimentally proven (superscripted) siRNAs calculated using MMPBSA approach.

Number	Ago2-siRNA complex	Energy components (kcal/mol)				Binding free energy (Kcal/mol)
		Vdw	Electrostatic	Solvation		
				Polar	Nonpolar	
1	128	−237.72	−5682.68	1243.344	−28.6209	−4702.53
2	251	−238.066	−5616.33	1197.62	−30.6692	−4684.31
3	341	−174.241	−5597.275	976.25	−24.0349	−4819.66
4	383	−201.869	−5449.78	974.5913	−25.7027	−4699.62
5	537	−238.258	−5613.16	1134.185	−30.0789	−4744.14
6	1113	−236.201	−5621.61	1151.83	−29.081	−4735.33
7	1115	−256.291	−5112.95	918.6902	−27.0244	−4474.58
8	ICAM2^exp⁡^	−231.954	−5754.27	1167.813	−29.254	−4846.78
9	GRK^exp⁡^	−209.262	−5451.12	1077.131	−26.292	−4610.42
