# Supplementary material for: Structural Dynamics of Human Argonaute2 and Its Interaction with siRNAs Designed to Target Mutant tdp43
Source: Adv Bioinformatics. 2016 Mar 6;2016:8792814. doi: 10.1155/2016/8792814 (PMC4824133; doi:10.1155/2016/8792814)

**Supplementary Figure 1.**
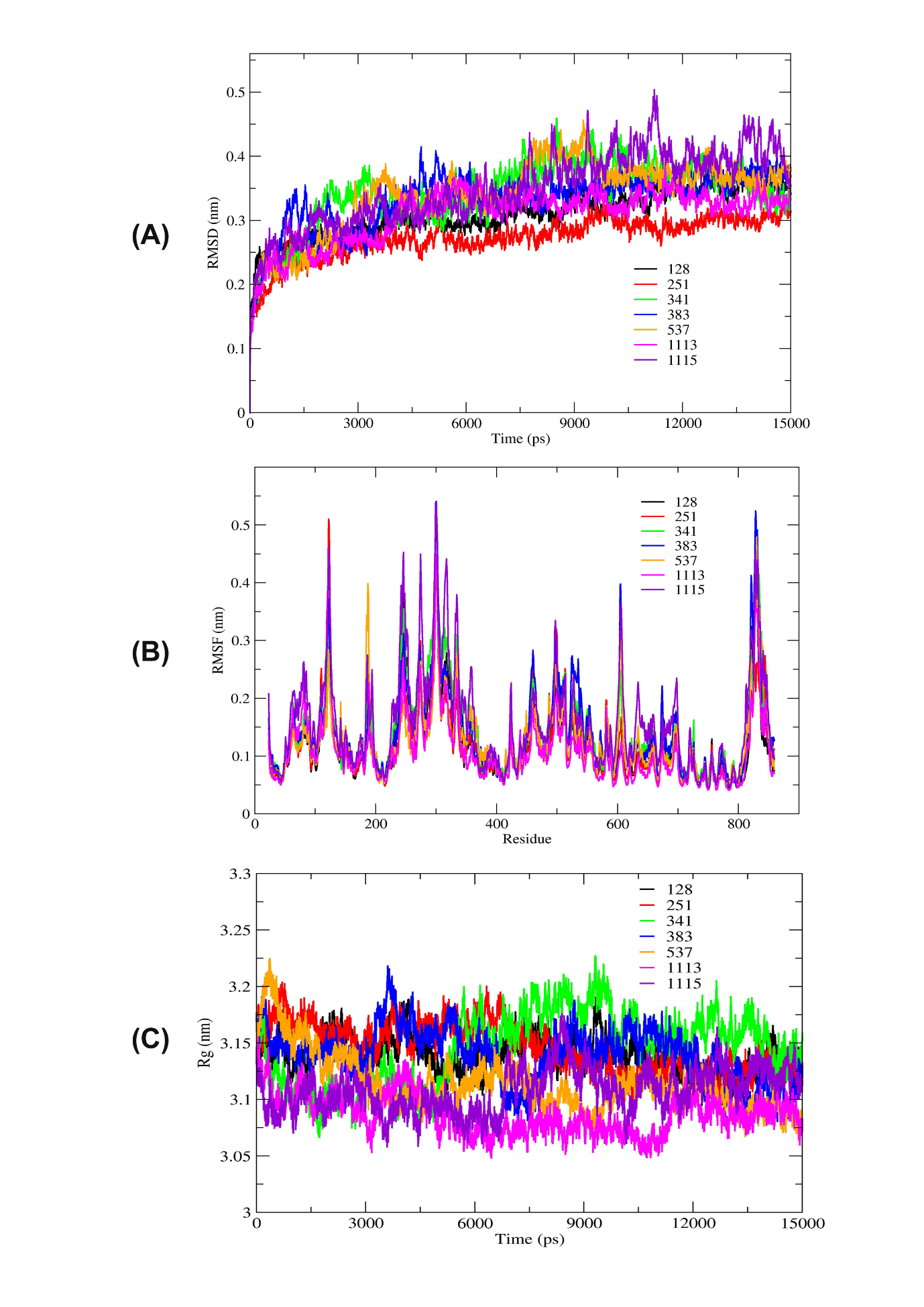
Variation in RMSD (A), RMSF (B) and Rg (C) values calculated during dynamics.

**
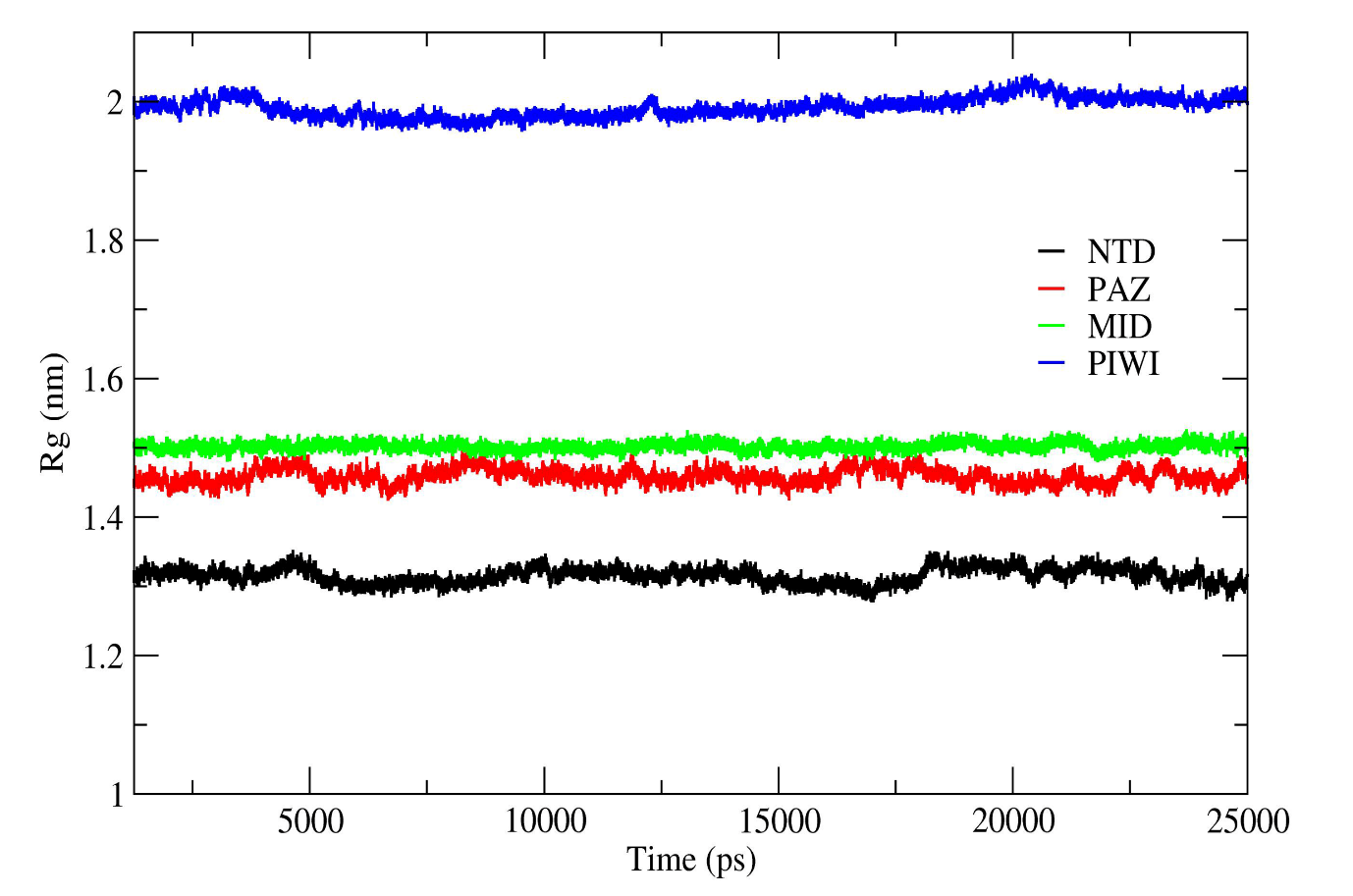
Supplementary Figure 2.** Radius of gyration of NTD, PAZ, MID and PIWI domains calculated for the free Ago2 during dynamics.

**
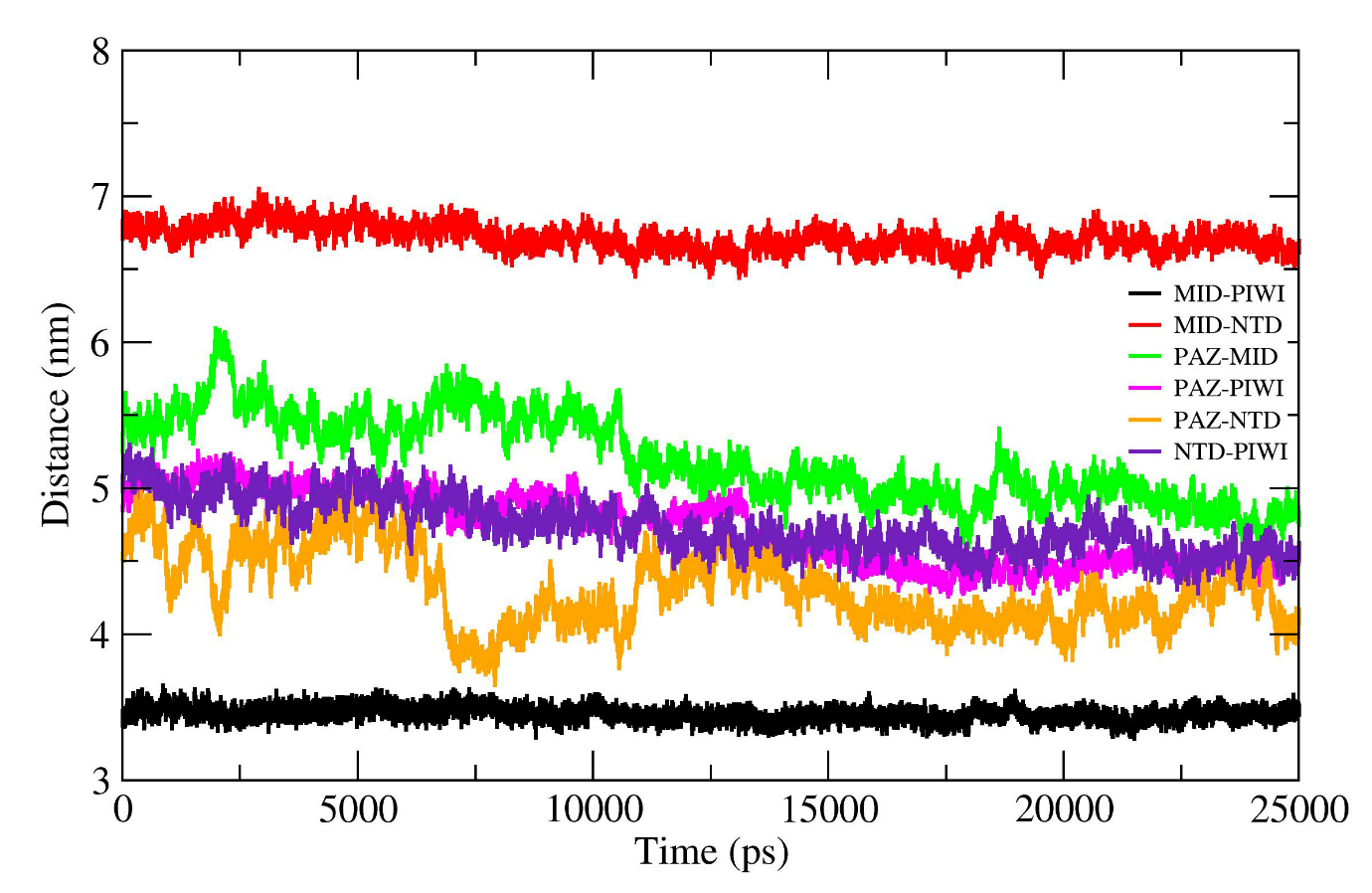
Supplementary Figure 3.** Inter domain distance in free Ago2 protein calculated during dynamics.


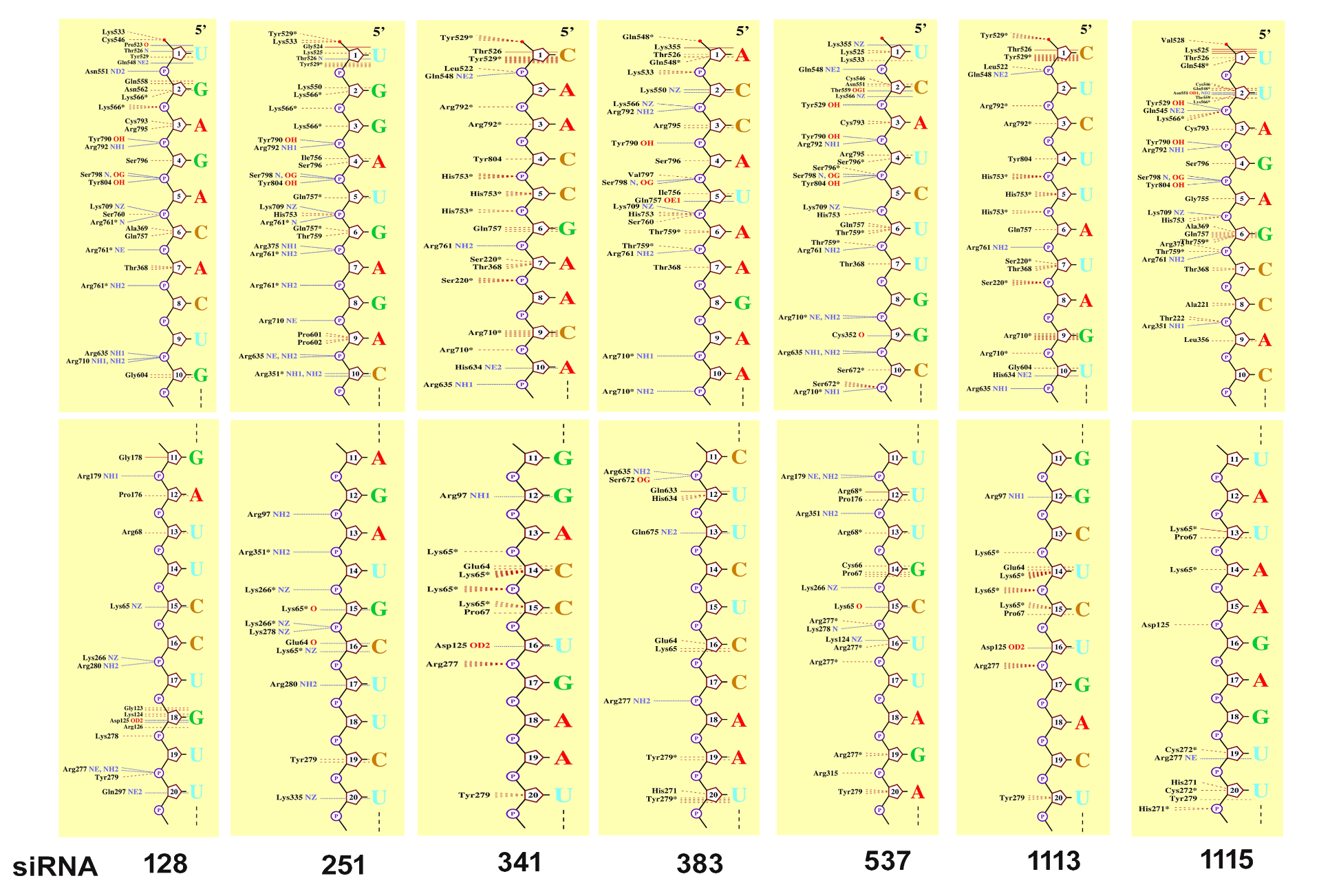
**Supplementary Figure 4.** Site-specific interactions observed between human Ago2 and all the studied siRNAs.

**
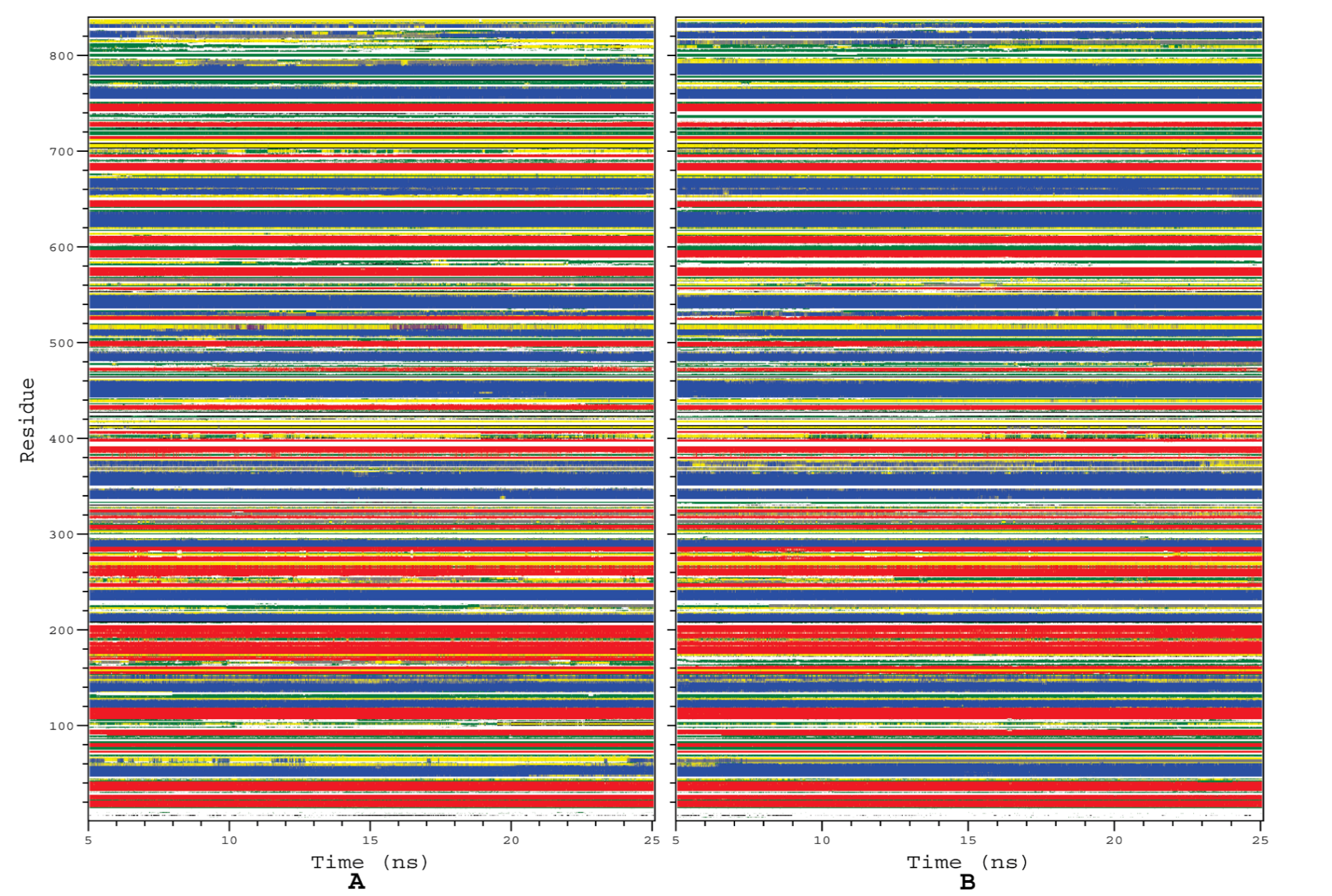
Supplementary Figure 5.** Changes in the secondary structure of free (A) and siRNA_341_ bound (B) Ago2 protein observed during simulation.

**Supplementary Figure 6.**  The open (grey) and close (cyan) conformations of human Ago2 protein superimposed with Ago2-siRNA_341_ complex (purple).


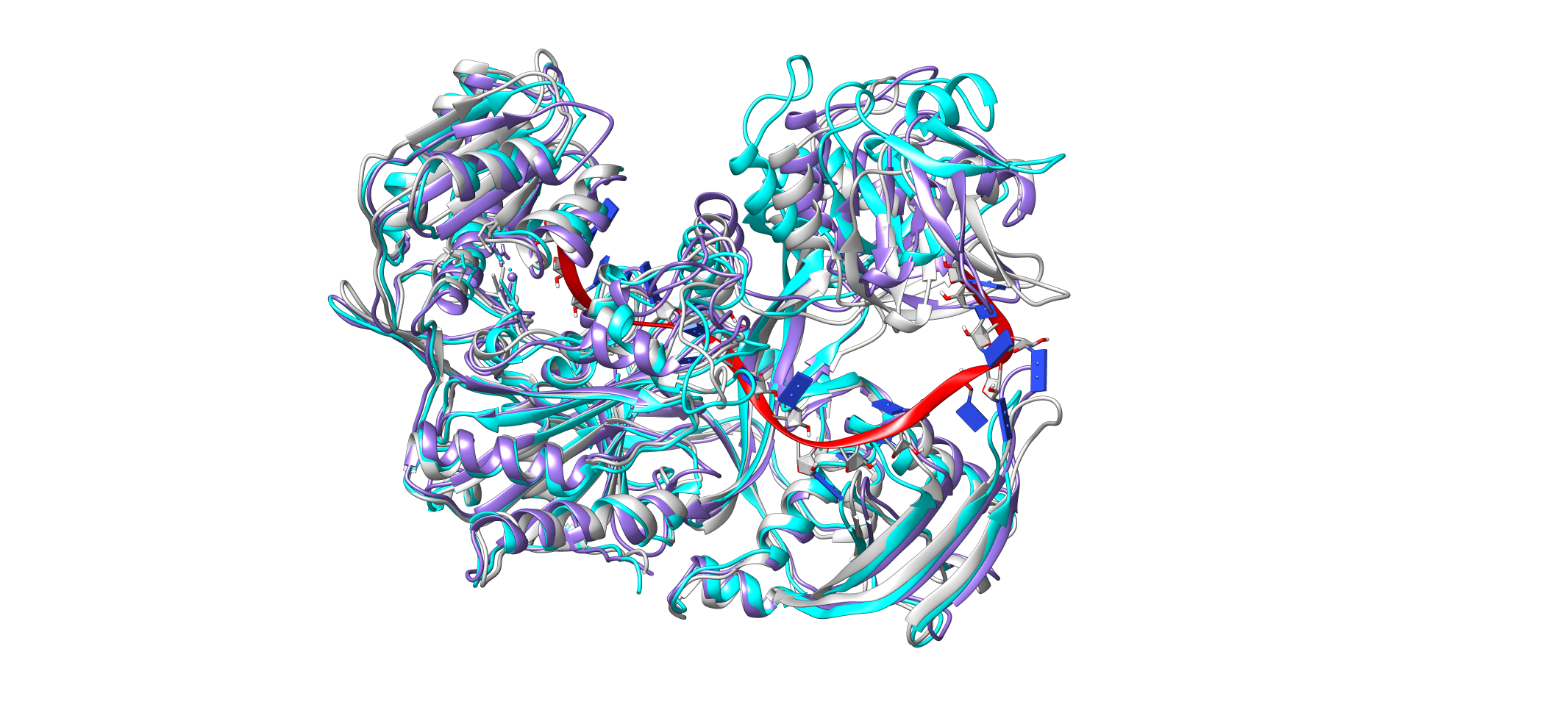


**Supplementary Figure 7.**  Superimposition of all siRNAs to depict their orientation inside human Ago2 protein after complexation


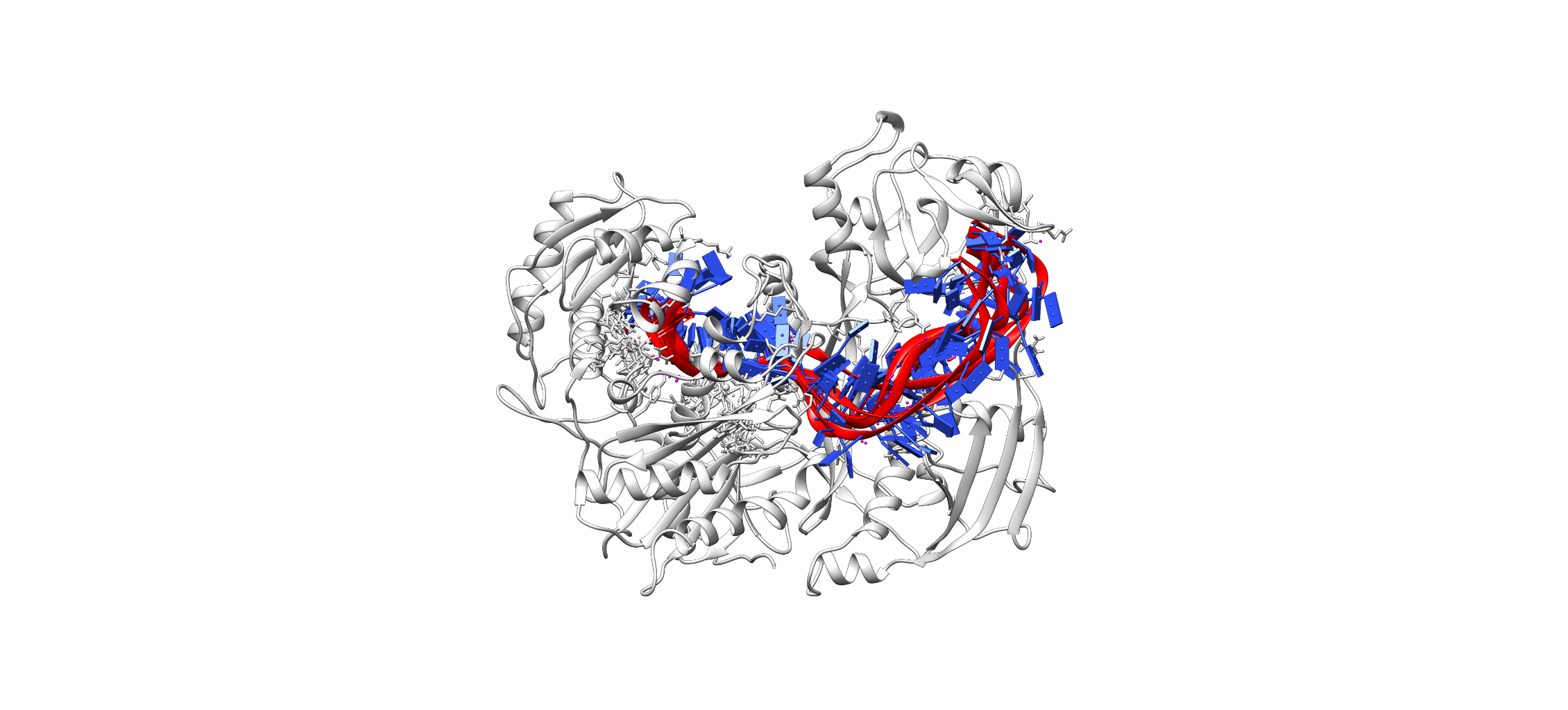

Supplement: Supplementary file 1 — Supplementary material contains total 7 figures which are highlighted in the manuscript. Supplementary Figures 1 and 2 represent the parameters explaining the structural stability observed during simulations. Supplementary Figures 3 and 4 show the inter domain distance in free Ago2 and site specific interactions between Ago2 and siRNA, respectively. Changes in the secondary structures are given in the supplementary Figure 5. Supplementary Figures 6 and 7 represent the three dimensional conformations. [file 8792814.f1.docx]
